# Boosting of Cross-Reactive and Protection-Associated T Cells in Children After Live Attenuated Influenza Vaccination

**DOI:** 10.1093/infdis/jix165

**Published:** 2017-03-27

**Authors:** Kristin G. I. Mohn, Fan Zhou, Karl A. Brokstad, Saranya Sridhar, Rebecca J. Cox

**Affiliations:** 1 The Influenza Centre,; 2 K. G. Jebsen Centre for Influenza Vaccines, and; 3 Broegelmann Research Laboratory, Department of Clinical Science, University of Bergen, and; 4 Emergency Care Clinic and; 5 Department of Research & Development, Haukeland University Hospital, Bergen, Norway; and; 6 Jenner Institute, University of Oxford, United Kingdom

**Keywords:** Influenza, LAIV, children, T-cell, cellular immune response, cross-reactive, heterologous, vaccine, protection.

## Abstract

**Background.:**

Live attenuated influenza vaccines (LAIVs) stimulate a multifaceted immune response including cellular immunity, which may provide protection against newly emerging strains. This study shows proof of concept that LAIVs boost preexisting, cross-reactive T cells in children to genetically diverse influenza A virus (IAV) strains to which the children had not been exposed.

**Methods.:**

We studied the long-term cross-reactive T-cell response in 14 trivalent LAIV–vaccinated children using the fluorescent immunospot assay (FluoroSpot) with heterologous H1N1 and H3N2 IAVs and CD8^+^ peptides from the internal proteins (matrix protein 1 [M1], nucleoprotein [NP], polymerase basic protein 1 [PB1]). Serum antibody responses were determined by means of hemagglutination inhibition assay. Blood samples were collected before vaccination and up to 1 year after vaccination.

**Results.:**

Preexisting cross-reactive T cells to genetically diverse IAV strains were found in the majority of the children, which were further boosted in 50% of them after receipt of LAIV. Further analyses of these T cells showed significant increases in CD8^+^ T cells, mainly dominated by NP-specific responses. After vaccination with LAIV, the youngest children showed the highest increase in T-cell responses.

**Conclusion.:**

LAIV boosts durable, cross-reactive T-cell responses in children and may have a clinically protective effect at the population level. LAIV may be a first step toward the desired universal influenza vaccine.

## Summary

This pediatric live attenuated influenza vaccine (LAIV) study is the first to show long-term, cross-reactive CD8^+^ T-cell responses to LAIVs. In the absence of preexisting antibodies, LAIV boosted preexisting T-cell responses to genetically diverse, wild-type IAVs to which the children were not previously exposed.

T cells play a key role in immunological responses in the control and clearance of viral infections. Both CD4^+^ and CD8^+^ T cells have been associated with less severe influenza in humans [1, 2]. Influenza remains among the most important respiratory viruses, and the World Health Organization estimates that 20%–30% of children and 5%–10% of adults are infected during an annual epidemic [3]. Vaccination is the most cost- effective strategy to combat the public health burden of influenza; however, current influenza vaccines require annual updating to cover new emerging strains, which constantly evolve to circumvent antibodies. In the absence of neutralizing antibodies, current inactivated influenza vaccines have poor efficacy against newly emerging influenza A viruses (IAVs), as was evident during the 2009 pandemic. In 2013–2014, substantial drift in the H3N2 virus caused a mismatch between circulating and vaccine strains, leading to excess mortality [4]. Thus, the challenge is to develop broadly cross-reactive vaccines that are protective in the face of constant viral evolution [5].

CD8^+^ T cells, an important T-cell subtype, are capable of killing virus infected cells by cytotoxic activity. Early work showed that cytotoxic CD8^+^ T cells play an important role in the recovery from influenza infection, in the absence of protective antibodies [6]. More recently, protection against disease and viral shedding was shown with cross-reactive CD4^+^ and CD8^+^ T cells specific to conserved internal viral proteins [2, 7].

Current inactivated influenza vaccines induce strain-specific antibodies and not CD8^+^ T cells [8]. However, live attenuated influenza vaccine (LAIV) mimics natural infection with viral replication in the upper respiratory tract and induces multifaceted immune responses, including antibodies and CD4^+^ and CD8^+^ T cells [8, 9]. Although LAIV elicits durable strain- specific T cells, whether cross-reactive CD8^+^ T cells are induced is less clear [8–10]. Studies have shown induction of CD8^+^ T cells after LAIV vaccination, but the kinetics and durability of these T cells was not reported [8, 11]. 

Partial protection through T cells may also have a clinical impact, reducing the severity of influenza-associated illness. Such evidence was found in a recent murine study, wherein LAIV was found to induce CD8^+^ T cells, which provided protection from heterosubtypic challenge [12]. Thus, a key question is whether LAIV is capable of inducing protection- associated cross-reactive T cells in humans. Furthermore, it is critical to know whether LAIV generates broadly protective T cells against novel strains to which individuals have no previous exposure. To address this question, we conducted a pediatric clinical trial in Norway, to test for influenza-specific cross- reactive T cells and determine their specificity and durability after immunization with trivalent LAIV.

## MATERIAL AND METHODS

### Study Subjects and Vaccine

Fourteen healthy children (median age, 4 years old; range, 3–15 years) were recruited in 2013–14 (October-February) and immunized with 1 (n = 4; ≥10 years old) or 2 doses (n = 10; <10 years old; 28-day interval) of trivalent LAIV (Fluenz) containing A/California/07/09(H1N1), A/Victoria-like/361/11(H3N2)/A/Texas/50/12(/H3N2) and B/Wisconsin/1/2010 strains. The study was a follow-up study, with a design identical to that of the previous season [9]. Healthy children and adolescents (3–18 years old) scheduled for elective tonsillectomy at the Ear-Nose and Throat Department at Haukeland University Hospital in Bergen, Norway, were eligible for inclusion. The exclusion criteria were the manufacturers contraindications for vaccination. All parents and children >12 years old provided informed consent before inclusion, and ethical and regulatory approval was obtained (www.clinicaltrials.gov; NCT01866540). 

Sequential blood samples were collected before and after vaccination, for up to 1 year. Peripheral blood mononuclear cells (PBMCs) were isolated using Cell Preparation tubes (CPT) and cryopreserved at −150°C in 90% fetal calf serum/10% dimethyl sulfoxide until used. Plasma samples were aliquoted and frozen at −80°C.

### Viruses and Peptides

Homologous, wild-type vaccine strains (A/California/07/09 [H1N1] and A/Victoria-like/361/11[H3N2]/A/Texas/50/12/[H3N2]) and heterologous, wild-type (A/Solomon Islands/ 03/06[H1N1] and A/Switzerland/9715293/13[H3N2]) strains were used to measure immune responses. Owing to limited blood volume, we tested only 1 heterologous virus per subtype. Synthetic, influenza-specific major histocompatibility complex class 1–restricted matrix protein 1 (M1), nucleoprotein (NP), and polymerase basic protein 1 (PB1) peptide pools were obtained from the BEI Resources, VA, USA.

### Hemagglutination Inhibition Assay

Plasma samples were analyzed in duplicate (starting dilution, 1:10) with 0.7% turkey red blood cells and 8 hemagglutinating units of the H1N1 and H3N2 homologous vaccine and heterologous wild-type viruses (50 μL per well). Negative samples were assigned a hemagglutination inhibition (HI) titer of 5 for calculation purposes [13].

### FluoroSpot Assay

Antigen-specific interferon (IFN) γ^+^, interleukin 2 (IL-2)^+^, and IFN-γ^+^/IL-2^+^ cytokine-secreting T cells were quantified at the single-cell level with the FluoroSpot assay (Mabtech) [14]. Briefly, PBMCs were thawed, and 400000 PBMCs per well for CD8^+^-conserved peptide pools (2 μg/mL) or 300000 PBMCs per well for live virus (multiplicity of infection, 5), anti-CD3 T-cell activator (positive control), or lymphocyte medium alone (negative control) were incubated overnight at 37°C. Spot-forming units (SFUs) were counted using an automated reader (Advanced Imaging Devices), and background values were subtracted.

## RESULTS

**Figure 1.**
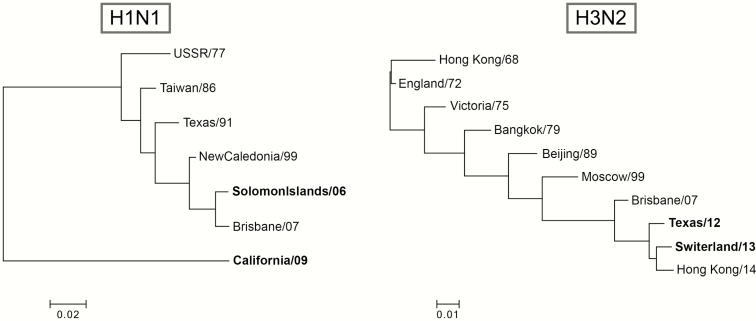
Phylogenetic trees for the influenza A H1 and H3 strains. The phylogenic trees show the genetic divergence from the homologous vaccine viruses and the heterologous wild-type strains. The influenza A/H1N1 strains were the California/09(H1N1) vaccine strain and the heterologous historical Solomon Islands/06(H1N1) strain from 2006. The influenza A/H3N2 strains were the homologous Texas/12(H3N2) live attenuated influenza vaccine (LAIV) vaccine strain and the drifted wild-type Switzerland/13(H3N2) strain from 2014–2015, the year after the trial. Phylogenetic trees were built using the neighbor-joining method with Poisson correction in MEGA software, version 6.0.6 [16].

In the current study, we examined the ability of LAIV to induce cross-reactive antibodies and T cells in children. To evaluate the immune responses to the heterologous H1N1 strain, we chose the historical Solomon Islands/06(H1N1) strain, which circulated 7 years before our study, and before 10 (71%) of the children were born. To evaluate the heterologous H3N2, we chose the Switzerland/13(H3N2) strain, which was antigenically distinct from the Victoria/12-like(H3N2)/Texas/12(H3N2) vaccine strain. This circulated in 2014–2015, the year after our study, so none of the children could have been exposed. There is a larger genetic distance between the H1N1 (0.22 in evolutionary distance) than the H3N2 strains (0.025 in evolutionary distance; Figure 1).

### Cross-Reactive Antibodies to Homologous and Heterologous Strains After LAIV

**Figure 2.**
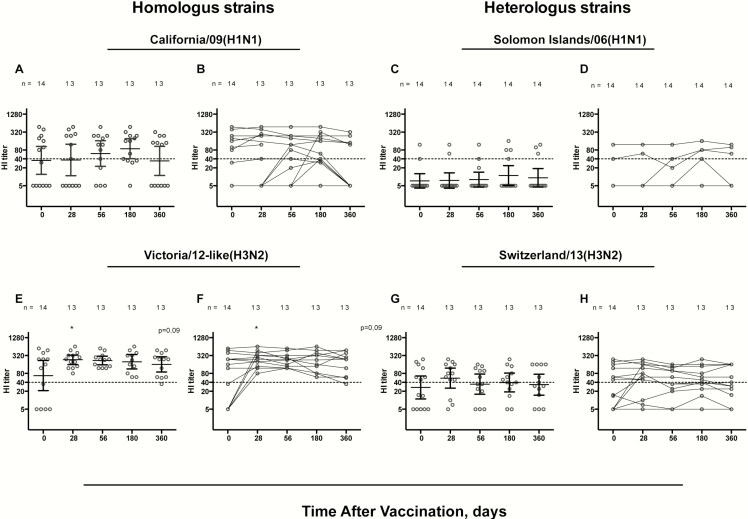
Hemagglutination inhibition (HI) responses to the homologous and heterologous influenza AHN1 and H3N2 strains. The HI responses after live attenuated influenza vaccine (LAIV) are shown for homologous vaccine strains, California/09(H1N1) (*A, B*) and Victoria/12-like(H3N2) (equivalent to Texas/12[H3N2]) (*E, F*) and the heterologous wild-type influenza strains, Solomon06(H1N1) (C, D) and Switzerland/13(H3N2) (G, H). Bars represent geometric mean titers with 95% confidence intervals,* indicating a trend of an increase (*P* = .09). The HI responses are shown as individual responses; each symbol represents a single child, and a titer of 40, considered the protective level, is indicated by dotted lines. To compare HI responses over time, the nonparametric Kruskal-Wallis test was used, with correction for multiple comparisons; differences were considered significant *at P* < .05.

We first assessed the vaccine strain-specific antibody elicited by LAIV. Before vaccination, HI antibodies above the protective threshold (HI titer, ≥40) were detected in ≥50% of children to the vaccine strains, in 7 and 8 of 14 children to California/09(H1N1) and Victoria/12-like(H3N2), respectively (Figure 2). All children reached HI titers of >40 for the Victoria/12-like(H3N2) strain, with a trend of an increase (*P* = .09) after 1 dose. Protective titers were maintained in 92% of children (12 of 13) at 1 year to the Victoira/12-like(H3N2), whereas only 3 children sero converted to California/09(H1N1) after LAIV, with 69% (9 of 13) having protective titers at day 56. When we studied the individual responses, all children with high prevaccination HI titers (≥40) maintained high titers (>80) up to 1 year after vaccination for both homologous vaccine strains, consistent with previous observations [9, 15].

Furthermore, we assessed HI antibodies to heterologous influenza strains. We detected before vaccination HI antibodies against the drifted Switzerland/13(H3N2) in 7 of 14 (50%) of the children. We found very high titers in 2 of 14 (14%) of the children (aged 11 and 13 years) against the more divergent Solomon Islands/06(H1N1) strain (Figure 2C and 2D). LAIV did not generally boost cross-reactive antibodies to either heterologous strain, but 3 of 14 children had a postvaccination increase in HI titers to the Switzerland/13(H3N2) (Figure 2G and 2H). There was no difference in HI titer fold change between the homologous and heterologous strains (Supplementary Figure 1).

### Boosting of Influenza-Specific T-Cell Responses to Cross-Reactive Strains After LAIV

We examined the vaccine strain-specific and cross-reactive T-cell responses after LAIV in the FluoroSpot assay. A level of 100 SFUs/10^6^ PBMCs has earlier been suggested to be protective after LAIV [17]. We observed prevaccination protective INF-γ^+^ T cells (>100 SFUs/10^6^ PBMCs) in 10 and 8 of 14 children in response to the vaccine California/09(H1N1) and Victoria/12-like(H3N2) strains, respectively. LAIV boosted the homologous T-cell response to the vaccine strains in 36%–50% of the children, 5 of 14 for California/09(H1N1) and 7 of 14 for the Victoria/12-like(H3N2) (data not shown).

**Figure 3.**
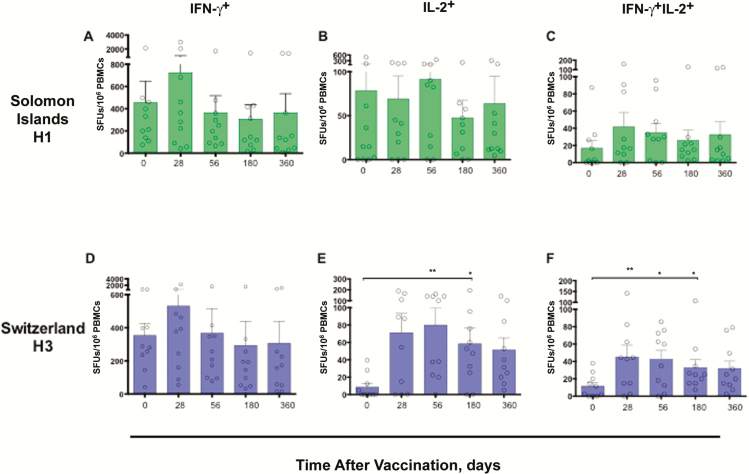
Durability of cross-reactive T-cell cytokine responses to heterologous influenza A virus strains after live attenuated influenza vaccine (LAIV). The long-term responses up to 1 y, for influenza-specific interferon (IFN) γ ^+^, interleukin 2 (IL-2)^+^, and IFN-γ ^+^IL-2^+^ T-cell responses after LAIV are indicated per 10^6^ peripheral blood mononuclear cells (PBMCs). Each symbol represents an individual child, and bars represents group means with standard errors of the mean. For statistical analysis of the heterologous T-cell response after vaccination, the nonparametric Kruskal-Wallis test was used, with correction for multiple comparisons; differences were considered significant at *P* < .05. * ; ** . Abbreviation: SFUs, spot-forming units.

To test whether LAIV induced cross-reactive T-cell responses, we measured influenza-specific responses to the wild-type historical Solomon Islands/06(H1N1) and the drifted Switzerland/13(H3N2) strains, which circulated the year after the study (Figure 3). High levels of preexisting IFN-γ^+^ T cells were also observed to the heterologous strains in most children, with 10 of 14 and 10 of 12 children with all time points available having levels >100 SFUs/10^6^ PBMCs for the Solomon Islands/06(H1N1) and Switzerland/13(H3N2) strains, respectively.

Remarkably, although 11 of 14 children showed no increase in HI antibodies to the heterologous Switzerland/13(H3N2) strain, a significant increase in IL-2^+^– and IFN-γ^+^IL-2^+^–secreting T cells was observed after LAIV. Importantly, these cross-reactive T cells were maintained for up to 6 months after vaccination. We found that the children either responded to or remained nonresponders to both the homologous Victoria/12-like(H3N2)/Texas/12(H3N2) (n = 7) and the heterologous Switzerland/13(H3N2) strains (n = 6) (Figure 3). 

For the more antigenically diverse (H1N1) strain, we observed a trend of an increase in IFN-γ^+^ T cells after 1 dose (day 28) (*P* = .08). However, when we examined the individual responses, 5 of 12 (naive) children who had no prevaccination antibodies to Solomon Islands/06(H1N1) showed increases in IFN-γ^+^ T cells in response to this older virus after 1 dose (Supplementary Figure 3). Looking at both strains, there was no significant difference in T-cell fold change between the homologous vaccine and the heterovariant strains (Supplementary Figure 2), indicating a response toward conserved viral proteins. Furthermore, for the individual immune responses, we observed that the children achieved either protective antibody titers (3 of 14 children), or high levels of T cells (71–957 SFUs/10^6^ PBMCs; 11 of 14 children) after LAIV [17]. 

### LAIV-Induced Increase in Protection-Associated, Cross-reactive CD8^+^ T Cells

**Figure 4.**
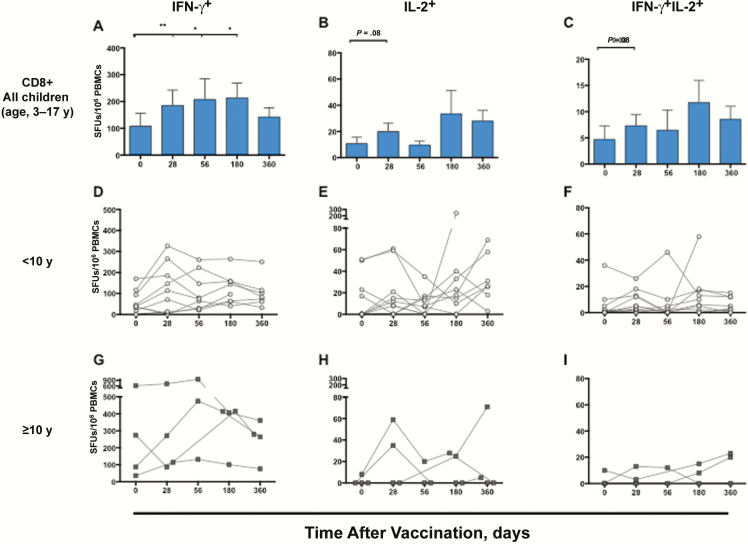
Long-term and individual influenza-specific CD8^+^ T-cell responses after live attenuated influenza vaccine (LAIV) vaccination. Influenza-specific interferon (IFN) γ ^+^, interleukin 2 (IL-2)^+^ and IFN-γ ^+^IL-2^+^ CD8^+^T-cell responses up to 1 y after LAIV are indicated per 10^6^ peripheral blood mononuclear cells (PBMCs). Bars represent means with standard errors of the mean. *A–C,* The IFN-γ ^+^ (*A*), IL-2^+^ (*B*), and IFN-γ ^+^IL-2^+^ (*C*) cytokine responses are shown for all children. *D–I,* The kinetics of the CD8^+^ T-cell response with individual responses are shown for children <10 y old (n = 10) (*D–F*) or ≥10 y old (n = 4) (*G–I*). For statistical analyses of the postvaccination response, the Wilcoxon matched-paired signed rank test was used for the 12 subjects with available samples up to day 180. * ; ** . Abbreviation: SFUs, spot-forming units.

To investigate whether the observed T cells were the protection- associated, cross-protective CD8^+^ T cells, we used CD8^+^-specific peptide pools in the FluoroSpot assay (Figure 4A–4C). Overall, the influenza-specific IFN-γ^+^ CD8^+^ cells showed the highest frequencies, throughout the study. Only 4 of 14 children had background levels of influenza-specific IFN-γ^+^ CD8^+^ T cells of >100 SFUs/10^6^ PBMCs, whereas 11 of 14 had levels of >20 SFUs/10^6^ PBMCs [7]. The IFN-γ^+^ responses increased significantly after 1 dose of LAIV (*P* < .01). For IFN-γ^+^ 11 of 14 children showed increases after 1 dose, 8 of 14 had levels >100 SFUs/10^6^ PBMCs, and 12 of 13 had an increase at day 56 compared with before vaccination. For IL-2^+^ and IL-2^+^/IFN-γ^+^ cells, there was a trend of an increase (*P* = .08), with an increase in 8 of 14 children (60%) at day 28. The IFN-γ^+^ cells were maintained up to 6 months, declining toward prevaccination levels within 1 year, whereas the long-term IL-2^+^ responses remained higher than prevaccination levels at days 180 and 360 (Figure 4A and 4B). Seven children (50%) had increases in all cytokine combinations after the first dose. Interestingly, we found a significant correlation between fold increases in CD8^+^ T cells and cross-reactive T cells after 1 dose (*P* = .01 for H1N1 and *P* = .01 for H3N2).

### Impact of Age and Priming Status on CD8^+^ T-Cell Responses

**Figure 5.**
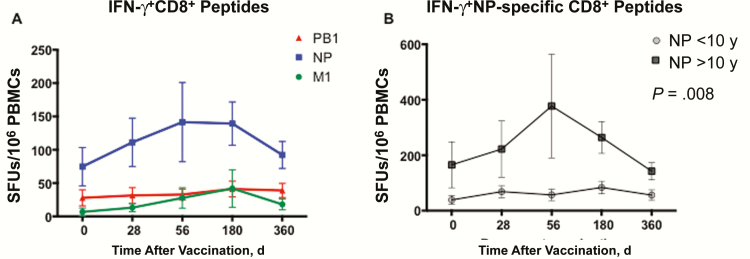
Influenza-specific individual CD8^+^ peptide responses after live attenuated influenza vaccine (LAIV) vaccination. *A,* Influenza-specific response to individual peptides covering the NP, M1, and PB1 proteins of influenza A virus, with means and standard errors of the mean. *B,* Because the dominant response was to the NP peptide, interferon (IFN) γ ^+^ NP-specific CD8^+^ peptide responses are shown according to age ≥10 or <10 y. The total response (NP, M1, and PB1) is shown in *A*. To compare the response over time between the 2 age groups, the nonparametric Mann-Whitney test was used, with differences considered significant at *P* < .05. Abbreviations: M1, matrix protein 1; NP, nucleoprotein; PB1, polymerase basic protein 1.

Because age determines the number of vaccine doses administered, we stratified the children’s CD8^+^ T-cell responses accordingly (Figure 4D–4I). Children ≥10 years old showed significantly higher CD8^+^ IFN-γ^+^ levels after vaccination (n = 4) than younger children (n = 10) (*P* = .008) (Figure 5), with increases after 1 dose in 3 or 4 older children. However, children <10 years old (n = 10), showed a trend of a significant increase in IFN-γ^+^ cells (*P* = .06) at day 28, with the majority (8 of 10; 80%) showing an increase after the first dose (Figure 4D–4F). Importantly, after the recommended second dose, all but 1 child <10 years old showed an increase from baseline. There was no overall correlation between age and fold change in CD8^+^ T cells after vaccination (data not shown). 

When we further studied the CD8^+^ T-cell response by the antibody priming status, of 10 children who were seronegative (HI titer, <10) to either homologous vaccine strain before vaccination, 9 had increases in IFN-γ^+^, IL-2^+^, or IFN-γ^+^IL-2^+^ CD8^+^ T cells after the first dose. We found no correlation between HI titers and the fold increase in IFN-γ^+^CD8^+^ responses at any time point (data not shown) or between HI titers before vaccination and CD8^+^T-cell fold change at day 28 (Supplementary Figure 3). Importantly, all serological nonresponders to California/09(H1N1) (HI titer of 5 at days 0–56) showed increases in CD8^+^ T cells. Interestingly, there was an overall increase in CD8^+^ T-cell numbers for the H1N1 strain in both seropositive and nonresponding children (Supplementary Figure 4).

### The T cell response to individual influenza proteins

To study the influence of each protein, the NP, PB1, and M1 responses were measured separately (Figure 5). Because the IFN-γ^+^ T cells dominated the response, we focused on this cytokine when studying the individual responses. We found the highest CD8^+^ responses to NP, with relatively low CD8^+^ levels to PB1 and M1 (Figure 5A). However, both the M1- and NP-specific CD8^+^ cells increased after the first dose. The NP response was significantly boosted (*P* = .02) after the second dose, maintained up to 6 months, followed by a decline, although the response remained above prevaccination levels. The combination of anti-NP and anti-M1 IFN-γ^+^ responses increased significantly only by days 28 and 56 (*P* = .03). When we further dissected the NP response, the effect of age became apparent. The children ≥10 years old showed significantly higher levels of anti-NP CD8^+^ T cells than those <10 years old (*P* = .008) (Figure 5B).

## DISCUSSION

Natural infection provides the basis for cross-reactive T cells, and historical studies provide evidence for heterovariant protection after influenza infection [1, 6, 18]. Current research toward the desired universal influenza vaccine includes focus on T cells and their potential for cross-protection across different IAVs. As opposed to inactivated influenza vaccines, the LAIV mimics natural infection, inducing broad T-cell responses [8, 9]. We hypothesized that these T cells could cross-react to heterovariant influenza strains. Our study found that up to 70% of children had preexisting cross-reactive, influenza-specific T cells in response to heterosubtypic IAVs in the absence of preexisting antibodies (70% to H1N1, 60% to H3N2). Furthermore, we found that the LAIV boosted T-cell responses to genetically diverse, wild-type IAVs, to which the children could not have been previously exposed and to which LAIV did not generally induce antibody responses.

Cross-reactive T cells are considered important in limiting disease severity and death in the absence of strain-specific antibodies, such as during a pandemic [2, 6]. It is noteworthy that the overall levels of preexisting heterovariant influenza-specific IFN-γ T cells were high, with most children having levels of >100 SFUs/10^6^ PBMCs before vaccination, which were boosted after receipt of LAIV. These findings indicate a considerable number of preexisting influenza-specific T cells with cross- reactive potential in our pediatric population, confirming findings at a population level (>20 SFUs/10^6^ PBMCs) [7]. 

These preexisting T cells may explain the diversity of clinical symptoms after infection, from asymptomatic to fulminant pneumonia [19]. Remarkably, in children without detectable HI antibodies to divergent strains, we found high levels of cross-reactive T cells in all but 2 children before vaccination. Importantly, our study found that LAIV boosted cross-reactive T-cell responses to historical and drifted wild-type IAVs not included in the vaccine. This important finding could imply a considerable protective effect at the population level if LAIV is administered to children. Indeed, signs of herd immunity, despite circulation of drifted influenza strains, as well as a reduction in child hospitalization rates, have been observed in the United Kingdom, where widespread LAIV vaccination of school children was conducted, [20, 2^1^].

A previous study by Mohn et al [9], the longest reported follow-up of cellular responses in LAIV vaccinated children, showed increased levels of T cells for up to 1 year. The results from the current study provide proof of concept that LAIV boosts cross-reactive and protection-associated CD8^+^ T cells. This important finding may help explain the clinical observation that LAIV provides protection from culture- confirmed illness in the absence of a boost in HI antibodies [22]. This is also supported by the correlation in CD4^+^ and CD8^+^ T-cell responses, but no correlation between the cellular and HI responses [8]. 

Similarly, we found no correlation between HI titer and CD8^+^ T-cell responses, and children with high HI titers showed no increase in CD8^+^ T-cells, while children with low HI titers showed and increase, indicating a response in the cellular or humoral immunecompartement, but not both (Supplementary Figures 3 and 4). One child (15 years old) responded in both the humoral and cellular compartments, and 1 (3 years old) did not respond in either compartment, highlighting the individual variation in human immune responses. However, because LAIV is dependent on viral replication to elicit immune responses, we cannot exclude the possibility that LAIV viruses are neutralized by preexisting antibodies. In agreement with our pediatric findings, a Russian LAIV study among young adults found cellular immune responses in all subjects without an increase in HI antibody titers [23]. In the Russian study, baseline CD4^+^ and CD8^+^ responses were predictive of post-LAIV cellular immune responses. Although the Russian LAIV is considered more immunogenic, it induced HI antibodies in ≤50% of the subjects, suggesting that the lack of HI response we found is more likely linked to the LAIV itself, rather than to the Russian or US/European LAIV.

Our study is the first to our knowledge to show long-term, cross-reactive CD8^+^ T-cell responses after LAIV vaccination, with the youngest children having the largest increases. Generally, the T-cell response after infection is thought to be rapid, and the peak was earlier than day 7 in adults during the 2009 pandemic [24]. Although we found significant increases 28 days after LAIV, larger increases may have been detected if we had sampled at an earlier time point.

Cross-reactive T-cell responses are directed primarily to conserved internal viral proteins (M1, NP, PB1) [1, 2]. CD8^+^ T cells have been found essential in limiting disease, and providing protection on heterosubtypic challenge in animal studies [1^2^, ^25^]. Moreover, human studies have shown that preexisting CD8^+^ and CD4^+^ T cells, specific for these conserved proteins, have been associated with lower viral shedding and less severe influenza disease [1, 2, 7]. Interestingly, lower levels of CD8^+^ T cells were observed in adults hospitalized with pandemic influenza disease, perhaps leaving them vulnerable to severe disease [26, 27]. Whether it is the magnitude or the functionality of the CD8^+^T-cell response that has a protective effect remains to be elucidated [28]. We found a significant increase in the CD8^+^ IFN-γ-response after LAIV, with the greatest response toward the NP protein, supporting the importance of including NP in broadly protective T-cell–inducing vaccines [7].

Our study is limited by its few subjects, and larger studies will therefore be needed to confirm our findings. Furthermore, we do not know the duration of the CD8^+^ T cells beyond 1 year, hence the need for repeated annual vaccination. However, if these CD8^+^ T cells provide protection from severe illness, LAIV could prove to be an important public health countermeasure in a situation with mismatch of epidemic and seasonal vaccine strains, or in the face of a novel pandemic virus, before a strain-specific vaccine is available. The finding that 50% of the children had increases in CD8^+^ T cells may be explained by the HLA genetic diversity, with the peptides perhaps not covering all HLA types.

It is debated whether LAIV is effective in older age groups. Children’s developing immune systems, combined with the impact of previous exposures to influenza, may influence the age-related response to LAIV. Our results show age-dependent differences in the response, supporting immunization of the youngest children with 2 doses, although most respond after the first dose. Importantly, our findings suggest that if a child has a CD8^+^ T-cell response, this will be cross-reactive. The single child that did not respond with CD8^+^ T cells after vaccination did not respond to either cross-reactive strain.

In contrast to cross-reactive T cells, only strain-specific antibodies neutralize the virus. However, a study in LAIV vaccinated children found an induction of cross-reactive HI antibodies to a drifted H3N2 strain, and the children who were vaccinated but nevertheless infected showed milder symptoms than infected controls [29]. Interestingly, although LAIV protected against a closely related H3N2 strain, recent epidemiological studies showed no sterilizing immunity against more antigenically divergent strains [19]. However, cross-reacting neutralizing antibodies have been found for closely related strains [30–32]. 

Overall, we did not find cross-reactive antibodies to the Solomon Islands/06(H1N1) strain, except in 2 older children, indicating earlier infection. However, 3 children responded to the drifted Switzerland/13(H3N2) strain, which has a closer genetic relationship to the Texas/12(H3N2) vaccine strain (Figure 1). The general lack of antibody response may also explain the failure of LAIV to provide sterilizing immunity in the 2013–2014 season, when the Switzerland/13(H3N2) variant emerged. However, we found durable vaccine, strain-specific antibodies 1 year after vaccination, with lower responses to the H1N1 than to the H3N2 strain, similar to findings during the 2012–2013 influenza season [9, 13]. This strain variability could be a result of the current H1N1 strain being less immunogenic than during earlier seasons, an issue being addressed by the vaccine manufacturer [33, 34] Currently, there is disparity between LAIV effectiveness data in the United States and in Europe. In the United States, the lack of effectiveness caused concern and withdrawal of the prior LAIV recommendation. In Europe, LAIV has been found to provide protection and is recommended. The reason for these differences is currently unknown, and investigation is ongoing, but it could be related to the vaccine, the viruses, or the population’s exposure history [20, 35].

Efficacy studies measure viral replication in the upper respiratory tract in patients with influenza like illness; cross-reactive T cells cannot provide sterilizing immunity but could limit severe disease. Perhaps an improvement in LAIV efficacy studies would be to divide vaccinated subjects who become infected into mild and severe infection groups, potentially illuminating a clinical protective effect. Indeed, a study in the United Kingdom found LAIV to reduce hospitalizations in children [20]. Norway does not have effectiveness data, but surveillance data for the study year indicated that approximately 60% and 30% of influenza infections were due to H1N1 and H3N2 strains, respectively [36].

In conclusion, our unique trial is the first to show long-term cross-reactive T cells elicited by LAIV in children. Importantly, our study found that these preexisting and protection- associated CD8^+^ T cells were detectable in young children in the absence of antibodies, which is proof of concept that the LAIV boosts CD8^+^ T-cell responses to conserved antigens. The responses were durable, indicating that a cellular immune response could possibly last through a whole influenza season. Although our findings were limited by small numbers, they support our hypothesis that the LAIV has the potential to provide cross-protective immunity to drifted and potentially heterovariant strains. Hence, it could possibly be a step toward the desired universal influenza vaccine.

## Supplementary Data

Supplementary materials are available at *The Journal of Infectious Diseases* online. Consisting of data provided by the authors to benefit the reader, the posted materials are not copyedited and are the sole responsibility of the authors, so questions or comments should be addressed to the corresponding author.

## Supplementary Material

Suppl_fig2_foldchange_HI

Suppl_fig_2_T_cellfoldchange_H1_H3

Supplementary_fig_3_CD8resp_by_HIrespondere_non_responders

Suppl_fig_4_correlation_HI_CD8

Figure_legends_w_supplementary

## Notes

***Acknowledgments.*** We thank the children and parents who altruistically participated in this study. We also thank the staff at the pediatric clinical trial unit and Ear Nose and Throat Department at Haukeland University Hospital and Sarah Lartey and staff at the Influenza Centre for their help with conducting the study. We especially thank Dr Othmar Engelhardt at the National Institute for Biological Standards and Control, South Mimms, United Kingdom, for providing the heterologous Switzerland/12(H3N2) strain and for valuable input to the study. We thank Astra Zeneca for recommendations and assisting with vaccine purchase and import.

The following reagent was obtained through the National Institutes of Health Biodefense and Emerging Infections Research Resources Repository, National Institute of Allergy and Infectious Diseases: peptide arrays, peptides for expected conserved major histocompatibility complex class I epitopes of influenza virus A proteins, NR-2667.

***Financial support.*** The study was funded intramurally by the Influenza Centre at the University of Bergen. The Influenza Centre is funded by the Ministry of Health and Care Services, Norway, the Norwegian Research Council Globvac program (grant 220670/H10), the European Union (UNIVAX 601738), and Innovative Medicines Initiative (IMI) (IMI115672 FLUCOP), Helse Vest, and the K. G. Jebsen Centre for Influenza Vaccines.

***Potential conflicts of interest.*** All authors: No reported conflicts. All authors have submitted the ICMJE Form for Disclosure of Potential Conflicts of Interest. Conflicts that the editors consider relevant to the content of the manuscript have been disclosed.
