# Supplementary figures and images for: Boosting of Cross-Reactive and Protection-Associated T Cells in Children After Live Attenuated Influenza Vaccination
Source: J Infect Dis. 2017 Mar 27;215(10):1527–35. doi: 10.1093/infdis/jix165 (PMC5461427; doi:10.1093/infdis/jix165)

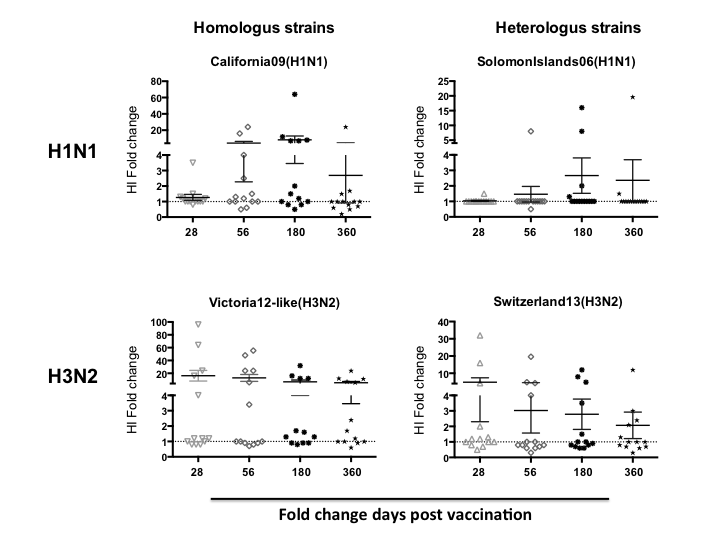

Supplement: Suppl_fig2_foldchange_HI [file jix165_suppl_Suppl_fig2_foldchange_HI.png]

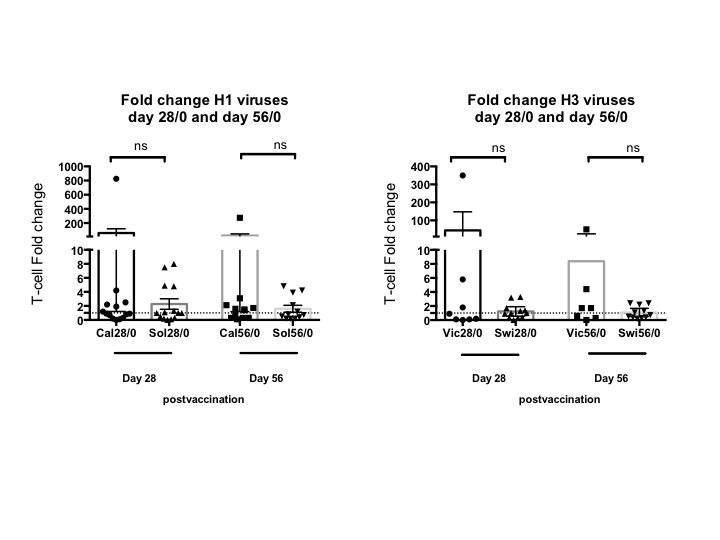

Supplement: Suppl_fig_2_T_cellfoldchange_H1_H3 [file jix165_suppl_Suppl_fig_2_T_cellfoldchange_H1_H3.png]

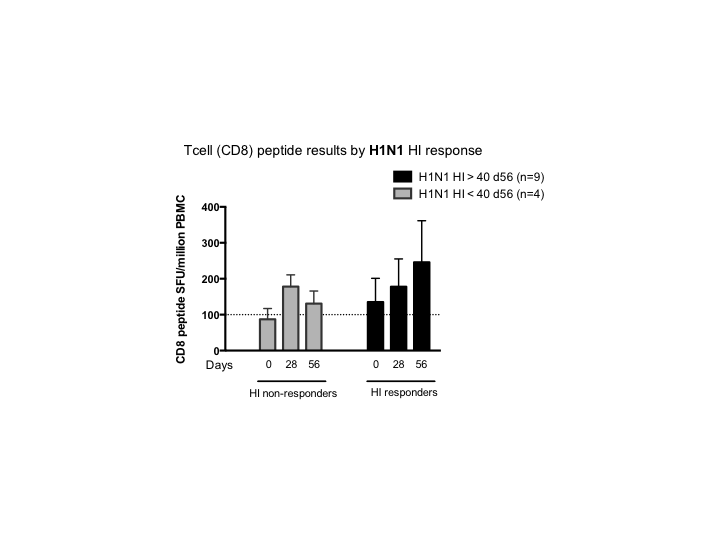

Supplement: Supplementary_fig_3_CD8resp_by_HIrespondere_non_responders [file jix165_suppl_Supplementary_fig_3_CD8resp_by_HIrespondere_non_responders.png]

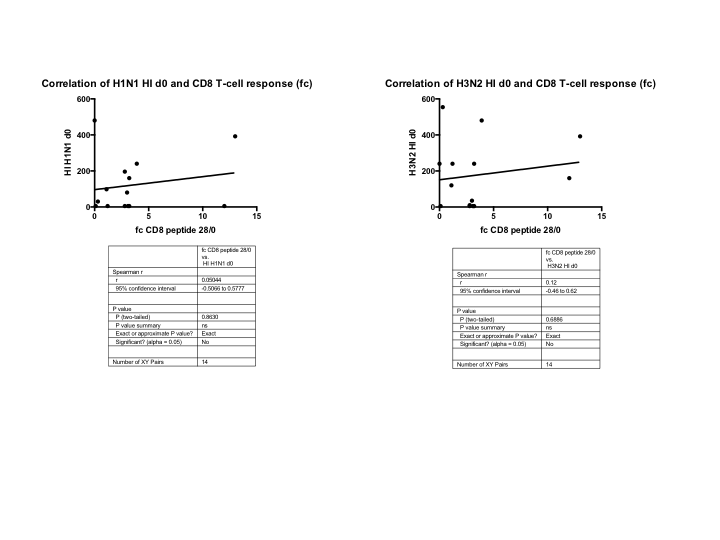

Supplement: Suppl_fig_4_correlation_HI_CD8 [file jix165_suppl_Suppl_fig_4_correlation_HI_CD8.png]
