# Supplementary material for: Boosting of Cross-Reactive and Protection-Associated T Cells in Children After Live Attenuated Influenza Vaccination
Source: J Infect Dis. 2017 Mar 27;215(10):1527–35. doi: 10.1093/infdis/jix165 (PMC5461427; doi:10.1093/infdis/jix165)
Supplement: Figure_legends_w_supplementary [file jix165_suppl_Figure_legends_w_supplementary.docx]

**Supplementary Figure 1: HI fold change for the homologous and heterologous influenza H1N1 and H3N2 strains.** Fold changes were calculated from pre-vaccination titers (day 0).

**Supplementary Figure 2:** **T-cell fold-change day 28 and 56 post vaccination to homologous and heterologous H1N1 and H3N2 strains.** The Wilcoxon matched-pair signed rank test was used to examine for statistical differences between homologous vaccine and heterologous strains. Fold changes were calculated from pre-vaccination titers (day 0).

**Supplementary Figure 3: CD8 peptide response after LAIV in children with and without an HI antibody response***.* Both responding children (HI<40) and non-responders (HI<40) after LAIV showed an increase in CD8 T-cell numbers.

**Supplementary Figure 4: Correlation between HI day 0 (pre-vaccination) and foldchange of the CD8 peptide response after one dose, day 28.** The non-parametric Spearman correlation test was used, with r indicated and with p<0.05 considered significant.
